# Faster Inversion Recovery Prepared T1 weighted segmented turbo field echo sequence (IR-TFE): Evaluating options for eliminating the start-up shot

**DOI:** 10.1186/1532-429X-13-S1-P28

**Published:** 2011-02-02

**Authors:** Ramkumar Krishnamurthy, Amol Pednekar, Benjamin Cheong, Raja Muthupillai

**Affiliations:** 1 Rice University, Houston, TX, USA; 2 Philips Health Care, Houston, TX, USA; 3 St. Luke's Episcopal Hospital, Houston, TX, USA

## Introduction

In conventional multi-shot IR-TFE sequences, e.g., clinical myocardial viability imaging, data collected during the first one or two shots is ignored, to minimize signal intensity variation across the shots. For a typical 8-10 heartbeat acquisition, such dummy shots results in scan overhead of 10-15% longer scan time. We evaluate the effect of removing the start-up shots in such IR-TFE sequences, and minimizing the effect of resulting artifacts

## Purpose

The purposes of this work are two fold: (a) to theoretically analyze and experimentally evaluate the impact of removing the startup shot in a conventional cardiac viability sequence, and (b) evaluate potential mechanisms for minimizing the artifacts stemming from the removal of start-up shots.

## Hypothesis

We hypothesize that by reducing the flip-angle of the IR pulse during the first shot one can minimize the signal amplitude modulation between the shots.

## Theory and methods

**Figure 1:**
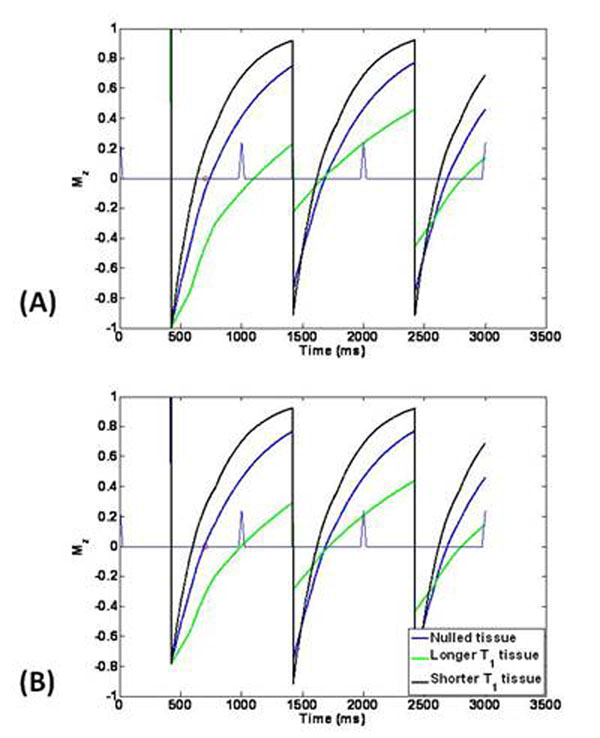


The magnetization preparation flip angle for the first shot was set to yield the steady state longitudinal magnetization for the tissue of interest. The variable flip angle was calculated as follows: With the user entered inversion time (TI) value, and the time between IR pulses, the apparent T1 of the tissue to be nulled is calculated for the given heart rate. Based on this T1, an inversion flip angle which would yield nulling at the desired Td for the first shot [Fig 1]. Each simulation was performed for three different tissue types that had apparent T1 values of 555 ms (tissue of interest), 1215 ms (longer T1) and 300 ms (shorter T1). Other simulation parameters were: TR/TE/α = 7 ms/3 ms/15°; TFE factor = 22; startup echoes = 4. The simulation was carried out for 7 RR intervals, each 1000 ms long, leading to 154 phase encoding steps. Phantoms with the desired T1 values (as simulated) were created using water and Magnevist® (Bayer Healthcare Pharmaceuticals, USA). Scanning was performed in a 1.5 T Achieva scanner (Philips Healthcare)

## Results

**Figure 2:**
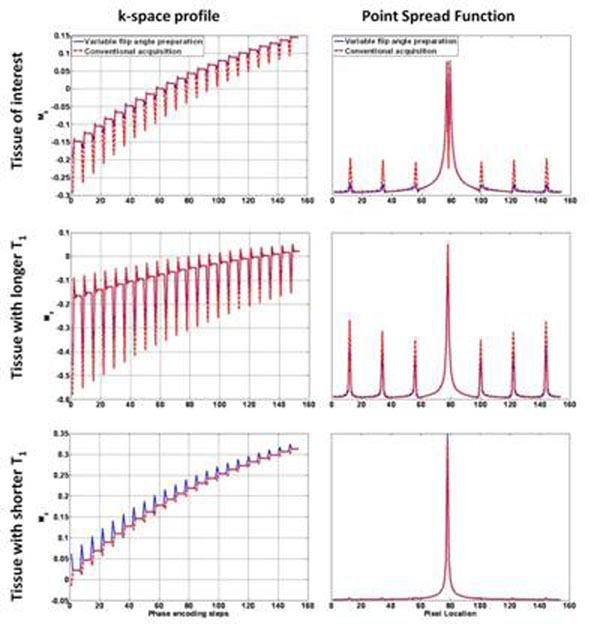


**Figure 3:**
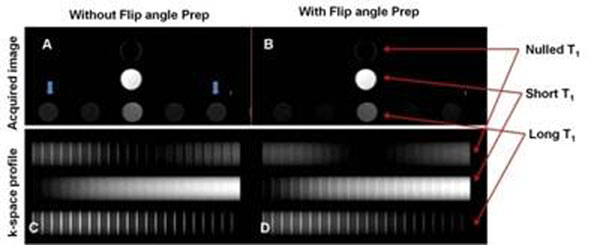


The theoretical point-spread function of IR-TFE sequence with/without the start up shots is shown in Figure 2. Experimental findings confirm theoretical predictions. Figure 3 shows the efficacy of the variable flip angle preparation method, which yields significantly less artifacts than the conventional method.

## Conclusions

Our theoretical and experimental models suggest that, by modulating the flip angle of the magnetization preparation pulse, it is possible to significantly reduce artifacts arising from accepting data from dummy shots.

